# Comment on “The Brazilian Portuguese version of the psoriasis epidemiology screening tool (PEST-bp) is reliable and accurate: a cross-sectional study from southern Brazil” – Reply^؆^

**DOI:** 10.1016/j.abd.2026.501370

**Published:** 2026-05-05

**Authors:** Vanessa Thomé, Marcia Regina Rosa Scalcon, Denise Teresinha Antonelli da Veiga, Luciane Prado de Vargas, Patrícia Chagas, Camila Sales Fagundes, Gabriel Caruso Novaes Tudella, Mateus Diniz Marques, André Avelino Costa Beber, Raíssa Massaia Londero Chemello, Diego Chemello

**Affiliations:** a Postgraduate Program of Health Sciences, Universidade Santa Maria, Santa Maria, RS, Brazil; b Department of Clinical Medicine, Division of Dermatology, Universidade Santa Maria, Santa Maria, RS, Brazil; c Department of Clinical Medicine, Division of Rheumatology, Universidade Santa Maria, Santa Maria, RS, Brazil; d Department of Public Health, Universidade Federal de Santa Maria, RS, Brazil; e Graduation, Universidade Santa Maria, Santa Maria, RS, Brazil; f Department of Clinical Medicine, Division of Cardiology, Universidade Santa Maria, Santa Maria, RS, Brazil

*Dear Editor,*

We read with great interest the comments on our article^1^ and appreciate the opportunity to further discuss and clarify our findings, which we believe helps to advance knowledge in this field.

First, regarding the use of a single static cutoff without stratified performance across specific patient subgroups, our primary objective was to assess the diagnostic accuracy of the PEST-BP in a new Southern Brazilian cohort using the same cutoff (≥3) proposed in the original English version and in the first Brazilian validation. Ibrahim et al. identified ≥3 as the optimal cutoff with high sensitivity and specificity, and Mazzotti et al. confirmed the same threshold in Brazilian patients.^2^, ^3^ In our sample, a score ≥3 yielded an AUC of 0.845 (p < 0.001), with 81% sensitivity, 79.7% specificity, and 80% overall accuracy, thus supporting the robustness of this cutoff in a distinct setting. As acknowledged in our discussion, the single-center design and relatively small sample size limit the power for reliable subgroup analyses (e.g., isolated nail psoriasis or early-onset disease), and we agree that future larger, multicenter studies should address stratified performance. Nonetheless, the PEST-BP already incorporates relevant PsA features, such as dactylitis and nail disease, and is intended as a simple, pragmatic screening tool for dermatology practice, in keeping with current recommendations favoring feasible questionnaires for PsA detection in routine care.^4^, ^5^

Second, concerning calibration between PEST-BP responses and actual joint involvement patterns, and whether dactylitis and nail psoriasis may have acted as confounders: in our study, patients with PsA had higher frequencies of dactylitis (38.1% vs. 11.4%; p = 0.004), nail psoriasis (66.7% vs. 35.4%; p = 0.01), and PASI ≥ 10 (42.9% vs. 19%; p = 0.023). However, in the multivariate logistic regression, only PEST-BP ≥ 3 and PASI ≥ 10 remained independently associated with PsA (OR = 32.43; p < 0.001 and OR = 9.26; p = 0.007, respectively), whereas nail psoriasis and dactylitis did not retain independent statistical significance. This supports that the predictive value of PEST-BP is not merely a reflection of these isolated manifestations, but of a broader composite of PsA-related symptoms and signs. PsA diagnosis was established using the CASPAR criteria, and PEST-BP performance was evaluated via ROC analysis against this established standard, which is an accepted approach for validating screening tools.^3^, ^6^ We agree that more granular calibration analyses linking specific PEST-BP response patterns to particular joint phenotypes would be valuable and should be pursued in larger, dedicated cohorts.

Third, regarding the inclusion of PASI ≥ 10 as an independent predictor and the possibility of conflating skin severity with joint risk, our results are consistent with evidence showing that higher psoriasis severity is associated with increased PsA risk. Eder et al. reported that severe psoriasis was a predictor of incident PsA in a prospective cohort, and Gelfand et al. observed higher PsA prevalence in patients with more extensive skin involvement.^7^, ^8^ In our multivariate model, PEST-BP ≥ 3 and PASI ≥ 10 were both independently associated with PsA, suggesting that they capture distinct but complementary dimensions of risk ‒ symptom-based screening versus cutaneous inflammatory burden ‒ rather than representing a simple overlap. We therefore view PASI ≥ 10 not as conflating the construct of the PEST-BP, but as an additive clinical parameter that may help prioritize rheumatologic referral, particularly among patients with moderate-to-severe disease. Future research may formally evaluate whether combined or stepwise strategies (e.g., PEST-BP plus PASI thresholds) can further optimize screening performance.

In conclusion, our study confirms that the PEST-BP with a cutoff ≥3 is a reliable and accurate screening tool for PsA in Brazilian dermatology settings, reinforcing its role in prompting timely rheumatologic evaluation rather than replacing specialist assessment. We are grateful for the constructive comments, which underline important directions for future research on subgroup performance and composite screening models.

## ORCID ID

Vanessa Thomé: 0009-0001-9335-4777

Denise Teresinha Antonelli da Veiga: 0009-0000-8507-4073

Luciane Prado de Vargas: 0000-0001-8591-9603

Patrícia Chagas: 0000-0001-9808-2187

Camila Sales Fagundes: 0000-0002-6895-0702

Gabriel Caruso Novaes Tudella: 0009-0001-4140-6045

Mateus Diniz Marques: 0000-0002-8160-2569

André Avelino Costa Beber: 0000-0001-8952-6073

Raíssa Massaia Londero Chemello: 0000-0002-6824-0962

## Financial support

This study was financed in part by the Coordenação de Aperfeiçoamento de Pessoal de Nível Superior – Brasil (CAPES) – Finance Code 001.

## Authors' contributions

Vanessa Thomé: Conceptualization; data curation; formal analysis; investigation; project administration; resources.

Marcia Regina Rosa Scalcon: Conceptualization; investigation; validation; project administration; writing-original draft.

Denise Teresinha Antonelli da Veiga: Conceptualization; validation; visualization; project administration; writing-original draft.

Patrícia Chagas: Software; validation; writing-review & editing.

Camila Sales Fagundes: Resources; software; writing-review & editing.

Gabriel Caruso Novaes Tudella: Resources; writing-review & editing.

Mateus Diniz Marques: Validation; visualization; writing-review & editing.

Luciane Prado de Vargas: Investigation; methodology; visualization.

Raíssa Massaia Londero Chemello: Resources; visualization; writing-review & editing.

Diego Chemello: Conceptualization; funding acquisition; methodology; supervision; writing-original draft.

## Research data availability

The entire dataset supporting the results of this study was published in this article.

## Conflicts of interest

None declared.
